# Management of metastatic breast cancer: are we prepared to cope with our own success?

**DOI:** 10.1038/sj.bjc.6602291

**Published:** 2004-12-14

**Authors:** A Jimeno, H Cortés-Funes, R Colomer

**Affiliations:** 1 Medical Oncology Division, University Hospital 12 de Octubre, Madrid, Spain; 2 Medical Oncology Division, Sidney Kimmel Comprehensive Cancer Center, Johns Hopkins University, Baltimore, MD, USA; 3 Medical Oncology Department, Institut Català d'Oncologia, Hospital Universitari Dr Josep Trueta, Girona, Spain

**Sir**,

In the first reported study to specifically define and address the issue of women presenting with stage IV breast cancer, we have recently evaluated the incidence and clinical course of initially metastatic breast cancer (IMBC) in a series of 1350 patients diagnosed and treated between 1992 and 1998 at our Institution (Jimeno *et al*, 2004). In this cohort, 8.9% of the patients presented with IMBC, a figure that is slightly greater than the reported incidence for stage IV breast cancer in the European Union (Sant, 2001), and higher than that reported in the analysis subject of this letter (Remak and Brazil, 2004). The fact that our data arises from a third-level Institution as opposed to population-based cancer registries, and that we classified as IMBC those cases in which metastases were detected in the first 3 months, may contribute to these differences.

It is very relevant for a cost analysis that we observed a median overall survival for the IMBC group of 25.1 months. These are to our knowledge the only published survival data in this subset of patients. The difference between this figure, and that used by Remák and Brazil (18 months, derived from Royal Marsden Hospital data), may therefore increase 40% the estimated cost of managing stage IV breast cancer. Thus, even if we consider that the true incidence of IMBC is 5%, the authors may have underestimated the real financial burden of IMBC management (£17 500 instead of £12 500).

Adding to the survival-related underestimation, Remák and Brazil calculated costs by assuming that 17 and 5% of patients received taxanes and trastuzumab, two of the most costly medications in our present therapeutic armamentarium. In a recent report assessing the patterns of treatment for metastatic cancer patients in Italy, taxanes were used initially in 46% of the patients (Cazzaniga *et al*, 2004). Despite ongoing controversies regarding which and when, the taxanes are being increasingly incorporated to the first-line setting, especially in anthracycline-resistant patients (Bernard-Marty *et al*, 2003), typically with four to six cycles. Regarding trastuzumab, there are not comprehensive reports indicating the proportion of patients who ultimately receive the antibody at some point during the course of their disease, but considering that approximately 20% of patients are HER2 positive, and that there is an increasing tendency to continue trastuzumab therapy despite disease progression (Gelmon *et al*, 2004), the reported estimates of 5% appear unrealistic. These figures would impact significantly what the authors considered the active phase of stage IV breast cancer. Assuming just four cycles of taxane-containing chemotherapy in 30% of patients with metastatic breast cancer would increase the tentative lifetime cost per patient in £3200.

Therefore, the cost of treating women presenting with stage IV breast cancer can be estimated to be at least £20 700. Increasing costs of advanced breast cancer should be put in context with overall population results end points, such as survival gains, which unfortunately are not usually reported separately for stage IV breast cancer.
